# Dynamic changes in human-gut microbiome in relation to a placebo-controlled anthelminthic trial in Indonesia

**DOI:** 10.1371/journal.pntd.0006620

**Published:** 2018-08-09

**Authors:** Ivonne Martin, Yenny Djuardi, Erliyani Sartono, Bruce A. Rosa, Taniawati Supali, Makedonka Mitreva, Jeanine J. Houwing-Duistermaat, Maria Yazdanbakhsh

**Affiliations:** 1 Department of Mathematics, Faculty of Information Technology and Science, Parahyangan Catholic University, Bandung, Indonesia; 2 Department of Biomedical Data Sciences, Leiden University Medical Centre, Leiden, The Netherlands; 3 Department of Parasitology, Faculty of Medicine Universitas Indonesia, Jakarta, Indonesia; 4 Department of Parasitology, Leiden University Medical Centre, Leiden, The Netherlands; 5 McDonnell Genome Institute at Washington University, St. Louis, Missouri, United States of America; 6 Department of Medicine, Washington University School of Medicine, St. Louis, Missouri, United States of America; 7 Department of Statistics, University of Leeds, Leeds, United Kingdom; University of Manchester, UNITED KINGDOM

## Abstract

**Background:**

Microbiome studies suggest the presence of an interaction between the human gut microbiome and soil-transmitted helminth. Upon deworming, a complex interaction between the anthelminthic drug, helminths and microbiome composition might occur. To dissect this, we analyse the changes that take place in the gut bacteria profiles in samples from a double blind placebo controlled trial conducted in an area endemic for soil transmitted helminths in Indonesia.

**Methods:**

Either placebo or albendazole were given every three months for a period of one and a half years. Helminth infection was assessed before and at 3 months after the last treatment round. In 150 subjects, the bacteria were profiled using the 454 pyrosequencing. Statistical analysis was performed cross-sectionally at pre-treatment to assess the effect of infection, and at post-treatment to determine the effect of infection and treatment on microbiome composition using the Dirichlet-multinomial regression model.

**Results:**

At a phylum level, at pre-treatment, no difference was seen in microbiome composition in terms of relative abundance between helminth-infected and uninfected subjects and at post-treatment, no differences were found in microbiome composition between albendazole and placebo group. However, in subjects who remained infected, there was a significant difference in the microbiome composition of those who had received albendazole and placebo. This difference was largely attributed to alteration of Bacteroidetes. Albendazole was more effective against *Ascaris lumbricoides* and hookworms but not against *Trichuris trichiura*, thus in those who remained infected after receiving albendazole, the helminth composition was dominated by *T*. *trichiura*.

**Discussion:**

We found that overall, albendazole does not affect the microbiome composition. However, there is an interaction between treatment and helminths as in subjects who received albendazole and remained infected there was a significant alteration in Bacteroidetes. This helminth-albendazole interaction needs to be studied further to fully grasp the complexity of the effect of deworming on the microbiome.

**Trial registration:**

ISRCTN Registy, ISRCTN83830814.

## Author summary

Studying the relationship between soil-transmitted helminthiasis and gut microbiota is becoming more important as both have been implicated in modulating immune system in various inflammatory diseases. However, findings of previous studies of the effect of helminth on the microbiome are inconsistent. In this study, an optimal design, a placebo-controlled anthelminthic trial was conducted to dissect the effect of helminths and anthelminthic treatment on gut microbial profile. In addition, a novel statistical model was used to analyse the association by taking into account the correlation structures between bacterial categories by applying multivariate analysis whereby the multiple testing correction is not needed.

## Introduction

Shortly after birth, the human body is colonized by a community of bacteria [1, 2] with relatively simple composition which increase in number and complexity with age [3]. The densest colonization with commensal microbes of the human body is found in the intestine [4] which has a beneficial impact on gastro-intestinal function and host health by providing support for host metabolism, protection against pathogenic microbes, integrity of intestinal mucosa, and modulation of the immune system [2, 3, 5]. Furthermore, it has been shown that intestinal microbiota is associated with dietary habits [6, 7], physiological factors such as age, gender and BMI [8, 9] as well as diseases, such as inflammatory bowel disease and obesity [1, 5, 10].

Apart from intestinal microbiota, certain pathogens such as soil-transmitted helminths (STH) may coexist in the human intestine. It is estimated that STH, largely represented by *Ascaris lumbricoides*, hookworm such as *Necator americanus* and *Ancylostoma duodenale*, and whipworm *Trichuris trichiura*, infect 2 billion people in the majority of developing countries and mostly children [1, 11]. These infections have been reported to cause impairments in physical, intellectual, and cognitive development [12]. At the same time, these parasitic worms have a long co-evolutionary interaction with their host. The result of this co-evolutionary trajectory, seems to be that helminths lead to immune regulatory responses that allow their long term survival within their host [13, 14]. Since intestinal microbiota and helminths share the same niche in their host, it is hypothesized that the presence or absence of intestinal helminths may affect their interaction with each other within the host. In an interesting study, evidence was provided for the beneficial effects of the microbiome on successful completion of whipworm life cycle [15]. Currently, there is also much interest to determine whether helminth infections affect the gut microbiome and whether the effects of worms on human health is mediated via alteration in the microbiome composition. It is becoming increasingly clear that the gut microbiota has important link to the immune system and several disease outcomes. With the mass drug administration programs underway to eliminate intestinal helminths in many endemic regions, it is essential to fully understand the consequences of deworming on community health by characterizing the effect on the gut microbial composition.

Recently, several studies investigated the relationship between the intestinal microbiome and intestinal helminth infections. In swines, a statistically significant association between *Trichuris* infection and the gut microbiome composition was shown [16, 17], evident from the altered abundance of the genus *Paraprevotella* and phylum Deferribacteres in the infected pigs. The chronic infection of *Trichuris muis* in C57BL/6 wild-type mice increased the relative abundance of Lactobacilli [18], while giving *T*. *trichiura* ova to macaques with chronic diarrhea increased the phylum Tenericutes and resulted in clinical improvement [19]. Therefore, in animal models, Trichuris infection seems to be associated with alternation in the gut microbiome. However, in humans, findings are not consistent. In an observational study in Ecuador, comparing the gut microbiome of infected and uninfected school children, no significant differences at various taxonomical levels were found [20]. On the contrary, two other observational studies in rural villages of Malaysia [21] and Zimbabwe [22] found a significant increase in diversity and abundance of certain bacteria taxa in infected compared to uninfected subjects. An increase in Paraprevotellaceae was seen in the Malaysian study, which seemed to be associated with *Trichuris* infection while an increase in *Prevotella* was reported in the study in Zimbabwe that was attributed to *S*. *haematobium* infection. Furthermore, in an interventional study carried out in another rural village in Malaysia [23], a significant change in order Bacteroidales and Clostridiales was observed after deworming while deworming of *S*. *haematobium* in an interventional study in Zimbabwe [22] did not seem to alter the microbiome.

The study designs which were used to investigate the human-gut microbiome in relation to helminth infections were either observational [20–22] or interventional without a control group [20–23] hampering the estimation of the true relationship between helminth infection and the microbiome composition. Motivated by the findings from previous studies of helminths on microbiome, we used samples from a larger randomized placebo-controlled trial of albendazole treatment in a population living in an area endemic for soil transmitted helminth infections [24] to further characterize the effect of helminth infection and treatment at before and 21 months after treatment. The study design allowed the investigation of the effect of helminths on the fecal microbial community through comparing helminth infected and uninfected at baseline and subsequently assessing the effect of treatment with albendazole. We also explored the effect of the interaction between treatment and infection status on the faecal microbiome. In addition, we used the opportunity to assess whether albendazole has a direct effect on the microbiome by analyzing those who received albendazole and were uninfected throughout the study. The placebo group enables the estimation of the effect of deworming on the microbiome composition in the absence of anthelminthic treatment which itself could affect the microbiome.

The analyses carried out in this study aim to characterize the joint effects of several predictors, such as helminth infection and treatment on each bacterial category. For comparing the gut microbiome of premature infants with different severities of necrotizing enterocolitis, a Dirichlet—multinomial model was used [25]. Here, we consider the same approach for modelling and hypothesis testing for the association between treatment and helminth infection on microbial composition at the phylum level. Our approach addresses the possible correlation between bacteria categories, the compositional feature of the microbiome data [26], and the multiple testing issue.

## Methods

### Ethics statement

This study was nested within the ImmunoSPIN study, a double blind placebo-controlled trial conducted in Flores Island, Indonesia [24]. The ImmunoSPIN study has been approved by the Ethical Committee of Faculty of Medicine, Universitas Indonesia, ref:194/PT02.FK/Etik/2006 and has been filed by ethics committee of the Leiden University Medical Center. The clinical trial was registered with number: ISRCTN83830814 in which the protocol for the trial and supporting CONSORT checklist are available elsewhere [27]. The subjects gave their informed consent either by written signature or thumb print. Parental consent was obtained for children below 15 years old.

### Sample populations and detection of soil-transmitted helminth (STH) infection

**Fig 1:**
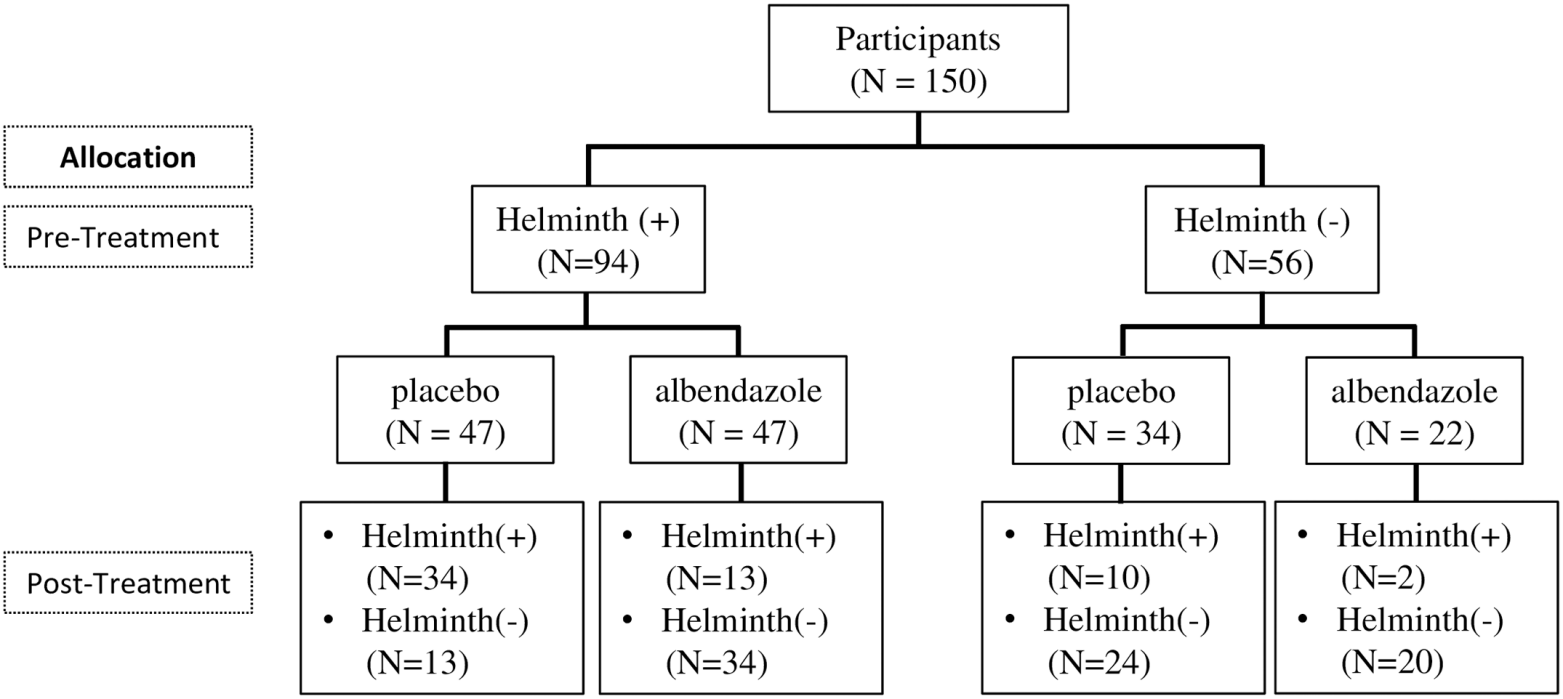
The profile of the microbiome study. The chart shows the number of subjecs infected with at least one of the prevalent soil transmitted helminths (Helminth (+)) or free of helminth infection (Helminth (-)) that belonged to either the placebo or albendazole treatment group, at pre-treatment and 21 months after treatment.

Households were randomized to receive either a single dose of 400 mg albendazole or placebo once every 3 months for 2 years. To assess the effect of treatment on the prevalence of soil transmitted helminth infection, yearly stool samples were collected on a voluntary basis. *T*. *trichiura* infection was detected by microscopy and a multiplex real time PCR was used for detection of hookworm (*A*. *duodenale*, *N*. *americanus*), *A*. *lumbricoides* and *Strongyloides stercoralis* DNA. For the current study, paired DNA samples before and at 21 months after treatment from 150 inhabitants in Nangapanda were selected based on the treatment allocation and infection status as well as the availability of complete stool data at pre and post-treatment (Fig 1). The procedure for sample collection and processing is already described elsewhere [24].

Briefly, prior to DNA isolations, approximately 100 mg unpreserved faeces (kept at -20°C) were suspended in 200μl PBS containing 2% polyvinylpolypyrolidone (PVPP;Sigma, Steinheim, Germany). Suspensions were heated at 100°C for 10 min and were treated subsequently with sodium dodecylsulphate-proteinase K at 55°C for 2 h. DNA was isolated using QIAamp DNeasy Tissue Kit spin columns (QIAgen, Venlo, The Netherlands). The whole procedure of DNA isolations and setup of PCR plates were performed using a custom-made automatic liquid handling station (Hamilton, Bonaduz, Switzerland).

As published already, sequences of the *A*. *lumbricoides* and *N*. *americanus*-specific primers and probes as well as the *A*. *duodenale* specific XS-probes were used to accommodate the specific fluorophor combinations of the CFX real-time PCR system (S1 Table) [24, 28]. The real-time PCRs were optimized first as monoplex assays with 10-fold dilution series of *A*. *duodenale*, *N*. *americanus* and *A*. *lumbricoides* DNA, respectively. The monoplex realtime PCRs were thereafter compared with the multiplex PCR with the PhHV internal control. The cycle threshold (Ct) values obtained from testing the dilution series of each pathogen in both the individual assay and the multiplex assay were similar, and the same analytical sensitivity was achieved.

Amplification reactions were performed in white PCR plates in a volume of 25μl with PCR buffer. Amplification consisted of 15 min at 95°C followed by 50 cycles of 15 s at 95°C, 30 s at 60°C, and 30 s at 72°C. Amplification, detection, and analysis were performed with the CFX real-time detection system (Bio-Rad laboratories). The PCR output from this system consists of a cyclethreshold (Ct) value, representing the amplification cycle in which the level of fluorescent signal exceeds the background fluorescence and reflecting the parasite-specific DNA load in the sample tested. In this manuscript, we set the ct value 30 as a threshold for the infection status i.e. subjects with PCR lower than 30 was identified as clearly infected and PCR above 30 as uninfected or very low infection. The analyses were carried with regard to the infection status and we do not consider the analysis in the level of infection.

### Sequencing of 16S rRNA gene

Genomic DNA samples were isolated from 100 mg of fresh stool, which were also used for detection of helminth infection by real time PCR. The DNA amplification and pyrosequencing followed the protocols developed by the Human Microbiome Project (HMP) [29] at the McDonnell Genome Institute, Washington University School of Medicine in St. Louis. Briefly, The V1-V3 hypervariable region of the 16S rRNA gene was amplified by PCR and the PCR products were purified and sequenced on the Genome Sequencer Titanium FLX (Roche Diagnostics, Indianapolis, Indiana), generating on average 6,000 reads per sample. The filtering and analytical processing of 16S rRNA data for this cohort has been previously described in details [30]. The assembled contigs count data as a result of RDP classification was organized in matrix format with taxa in columns and subjects in row. The entries in the table represent the number of reads for each phyla for each subject. Rarefaction to 2000 reads was performed using an R package (vegan) [31]. We obtained the count data of 609 bacterial genera and 18 bacteria phyla. In the analysis at phylum level, we retained the 5 most prevalent phyla (Actinobacteria, Bacteroidetes, Firmicutes, Proteobacteria, and Unclassified Bacteria) and pooled the remaining phyla into a pooled category such that there are only 6 phyla categories. The Unclassified bacteria represents the category where all the sequences cannot be assigned into a phylum. We conducted further analyses by decomposing the statistically significant phylum (Bacteroidetes) into the two most prevalent genera (*Bacteroides* and *Prevotella*) and the remaining genera into a pooled Bacteroidetes category and combining the Proteobacteria, Unclassified Bacteria and Pooled in a Pooled Phyla category. In total we have six categories since we also selected Actinobacteria and Firmicutes at phylum level.

### Statistical methods

The within sample diversity (Shannon and richness diversity) indices as well as the between sample diversity (Bray-Curtis distance) were computed at baseline and follow-up using the dataset at genera level. Clustering of samples and bacteria was studied by plotting a heat map of bacteria genera which were present in at least one sample and which had an average relative abundance of more than 1%. This cutoff was chosen to exclude rare genera. Unless stated otherwise, the rest of the analyses were done at the phylum level. A Pearson’s chi-squared test statistic was used to test for differences of infection prevalence between the two treatment groups at pre and at post-treatment.

Although the study design allows for the pairwise analysis, unfortunately no method is available for multivariate categorical count data. For this reason, we used the Dirichlet-multinomial regression where the characterization of infection and treatment are similar to the interpretation in loglinear model. Each count outcome within a category was assumed to follow the negative binomial distribution. This distribution is the result of a Poisson distribution for counts with the additional assumption that the underlying parameter is a random variable which follows the conjugate distribution (Gamma). By assuming that the underlying parameter was random, the presence of overdispersion due to multiple counts observed within a sample was modelled. To incorporate the fact that the total count is fixed per sample, we conditioned the probability of the multivariate count outcome on the total count per sample. This model is equivalent to the approach of Guimaraes and Lindrooth [32], i.e. the Dirichlet-multinomial regression model. The model parameters are log of odds ratios which compare the prevalence rate of each bacteria phyla associated with the covariates with the reference category. In all analyses, Firmicutes was used as reference since it has the highest abundance among the phyla. The covariates were infection status and treatment allocation which are both binary variables.

The likelihood ratio statistic was used to test the null hypothesis of no effect of the covariate on the microbiome composition. The test statistic follows asymptotically a *χ*^2^ distribution with *J* degrees of freedom, representing the *J* − 1 bacterial comparison with the reference and one overdispersion parameter.

As the Dirichlet—multinomial regression is available for cross-sectional setting, we modelled the association between microbiome composition and covariates including treatment at 21 months after treatment. First, we modelled the association between treatment and microbiome composition by including all study participants. Next, we selected subjects who were infected with at least one single helminth at baseline and included a categorical variable representing the four combinations of treatment allocation and infection status at post-treatment in the model. The R package MGLM [33] was used for analyses. The results were reported in terms of odds ratios, 95% confidence intervals and *p*-values.

To confirm our finding with this method, we used the univariate pairwise analysis for single bacterial categories of interest in albendazole arm. For this purpose, the inverted beta binomial test was applied to test the null hypothesis that the relative abundance of certain bacteria category at pre-treatment is similar to the relative abundance at post-treatment. Note that the inverted beta-binomial regression model is only defined for two categories and is equivalent to the Dirichlet-multinomial. The R package ibb [34, 35] was used for this test. All computations were conducted in R version 3.1.0 [36].

## Results

### Characteristics of the study subjects

At baseline, 94 out of 150 (62.7%) individuals were infected with one or more helminth species, and hookworm was the most dominant species (52.1%) followed by *T*. *trichiura* (44.7%) and *A*. *lumbricoides* (37.2%). The baseline characteristics such as age, gender, and helminth prevalence were similar between the two treatment arms although the prevalence of *N*. *americanus* was slightly higher in albendazole group, but not statistically significant (Table 1). The additional relevant characteristics of the participants are listed in S2 Table. With regard to the microbiome composition, the proportions of each bacterial phyla were also similar between two treatment arms with the highest abundance at the phylum level being Firmicutes followed by Actinobacteria, Proteobacteria and Bacteroidetes.

**Table 1:** Characteristics of the study subjects at baseline and at 21 months post-treatment.

Characteristics	pre-treatment		post-treatment	
	Albendazole arm	Placebo arm	Albendazole arm	Placebo arm
	(N = 69)	(N = 81)	(N = 69)	(N = 81)
Age (in years), mean (SD)	27.38 (16.5)	27.85 (16.9)		
Sex, female, n(%)	39 (56.5)	45 (55.6)	39 (56.5)	45 (55.6)
**Helminth Infections, n(%)**				
**Single infection**				
*A*. *lumbricoides*	17 (24.6)	18 (22.2)	1 (1.4)	7 (8.6)
Hookworm	26 (37.7)	23 (28.4)	3 (4.3)	11 (13.6)
*N*. *americanus*	25 (36.2)	23 (28.4)	3 (4.3)	10 (12.3)
*A*. *duodenale*	2 (2.9)	2 (2.5)	0 (0)	1 (1.2)
*T*. *trichiura*	20 (28.9)	22 (27.2)	9 (13.0)	9 (11.1)
**Multiple infection** ^•)^				
*A*. *lumbricoides*	17 (24.7)	18 (22.2)	3 (4.3)	23 (28.4)
Hookworm	26 (37.7)	23 (28.4)	3 (4.3)	20 (24.7)
*T*.*trichiura*	20 (28.9)	22 (27.1)	11 (15.9)	23 (28.4)
**Any helminth**	47 (68.12)	47 (58.0)	15 (21.7)	44 (54.3)
**Proportion (in %) of the 6 most abundant bacteria phyla, mean(SD)**				
Actinobacteria	12.5 (8.9)	11.0 (7.9)	13,2 (8.4)	11.8 (8.5)
Bacteroidetes	7.4 (11.3)	6.4 (11.0)	5.7 (9.5)	6.2 (12.5)
Firmicutes	66.8 (13.5)	70.0 (13.7)	66.0 (13.8)	68.1 (14.2)
Proteobacteria	9.8 (7.9)	9.2 (8.4)	11.7 (11.0)	10.1 (8.6)
Unclassified*^)^	2 (2.22)	2.7 (3.2)	2.1 (1.6)	2.6 (2.7)
Pooled^#)^	1.5 (3.7)	0.7 (1.2)	1.3 (2.2)	1.2 (2.4)

^•)^ Species is indicated that is in combination with one or more of the other helminth species.

*^)^Unclassified represents sequences that cannot be assigned to a phyla.

^#)^Pooled category consists of the remaining 13 phyla having average relative abundance among samples less than 1%.

**Fig 2:**
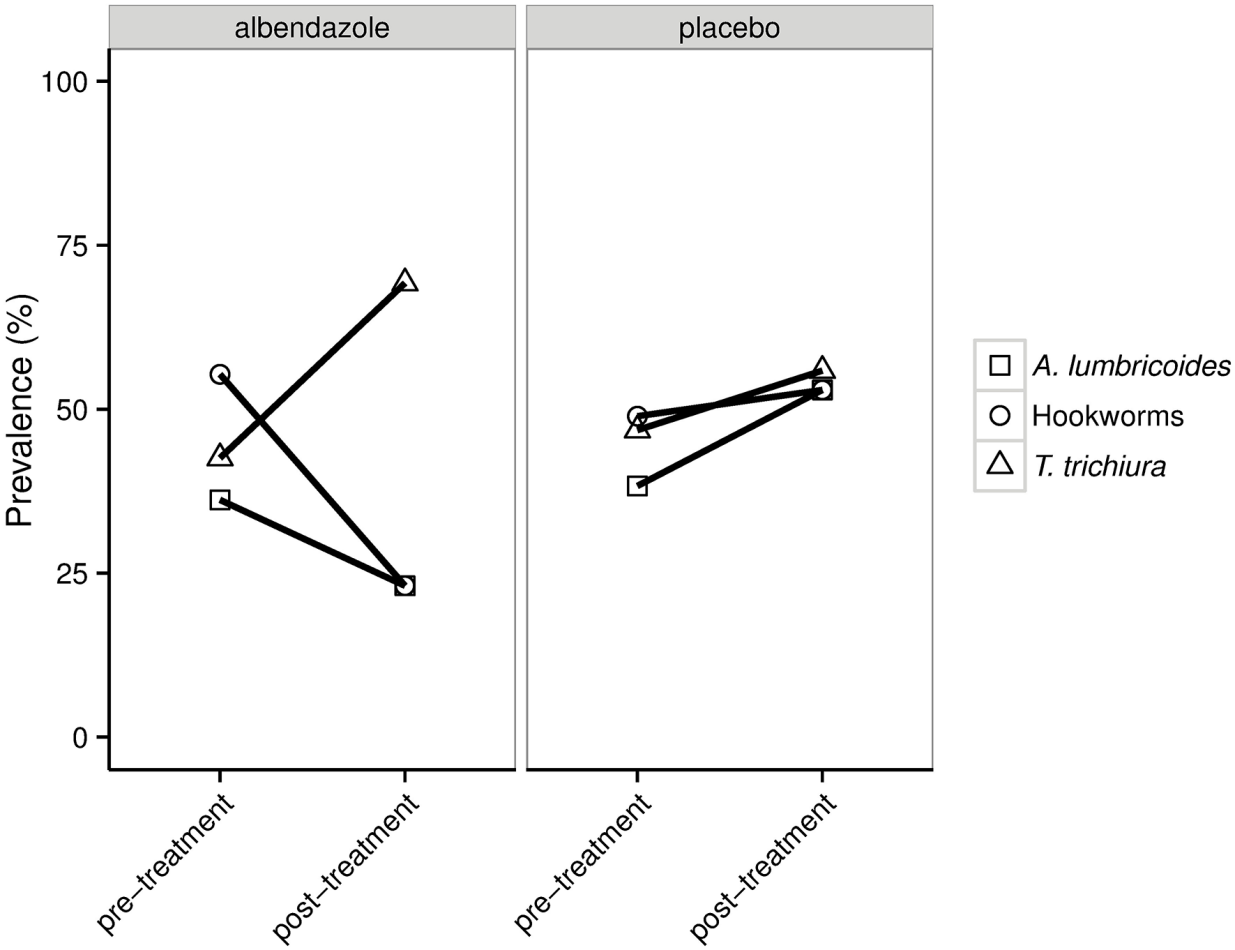
The prevalence of helminth coinfections in two randomization arms for subjects who were infected at pre-treatment and remained infected at post-treatment. For each helminth species depicted in the plot, square represents the percentage of subjects infected with *A*. *lumbricoides* (with or without other helminth species), circle represents hookworm (with or without other helminths) and triangle represents *T*. *trichiura* (with or without other helminths).

At 21 months after treatment, the prevalence of STH infection was 21.7% in the albendazole arm and 54.3% in placebo arm (*p*-value < 0.001). Albendazole had the greatest effect on hookworm (24.7% (placebo) vs 4.3% (albendazole)) followed by *A*. *lumbricoides* (28.4% (placebo) vs 4.3% (albendazole)) and lastly *T*. *trichiura* (28.4% (placebo) vs 15.9% (albendazole)). These percentages are similar to what was seen in the whole ImmunoSPIN trial [24]. These data show that while infections with *A*. *lumbricoides* and with hookworms decrease at post-treatment, the infections with *T*. *trichiura* was not affected much by albendazole and therefore the proportion of individuals infected with *T*. *trichiura* increased when considering those that remained infected at post-treatment (Fig 2). In the placebo group, there was no such difference in the composition of helminth species at post-treatment. It was noted that 12 (2 from albendazole and 10 from placebo) out of 56 uninfected subjects at baseline (21.4%) gained helminth infection over the study time period.

### Effects of helminths and treatment on microbiome diversity

Using bacterial data at the genus level (a total of 609 genera), we calculated the within sample diversity (richness and Shannon index) and between sample diversity (Bray-Curtis dissimilarity). We observed a similar within-sample diversity at pre and post-treatment as evident from the Shannon diversity index (2.99 vs 2.96) and the richness index (66.17 vs 62.16). The Bray-Curtis dissimilarity measures the percentage of similarities between two samples in a community and the values range from 0 (completely similar) to 1 (completely dissimilar). As reported earlier [30], the Bray-Curtis dissimilarities calculated from 150 subjects at pre-treatment was 0.61 and the same average was obtained when calculating the Bray-Curtis dissimilarities at post-treatment, indicating that in average there was 61% dissimilatory percentages between each pairs of samples. When stratifying all samples based on infection status at pre-treatment and on randomization arm at post-treatment, again we observed similar beta-diversities, indicating that neither infection nor treatment induced a shift in diversity. When analyzing the genera in relation to infection status rather than treatment, the average Shannon diversity index as well as the average richness was similar between the infected and the uninfected group at pre-treatment and post-treatment (S1 Fig).

**Fig 3:**
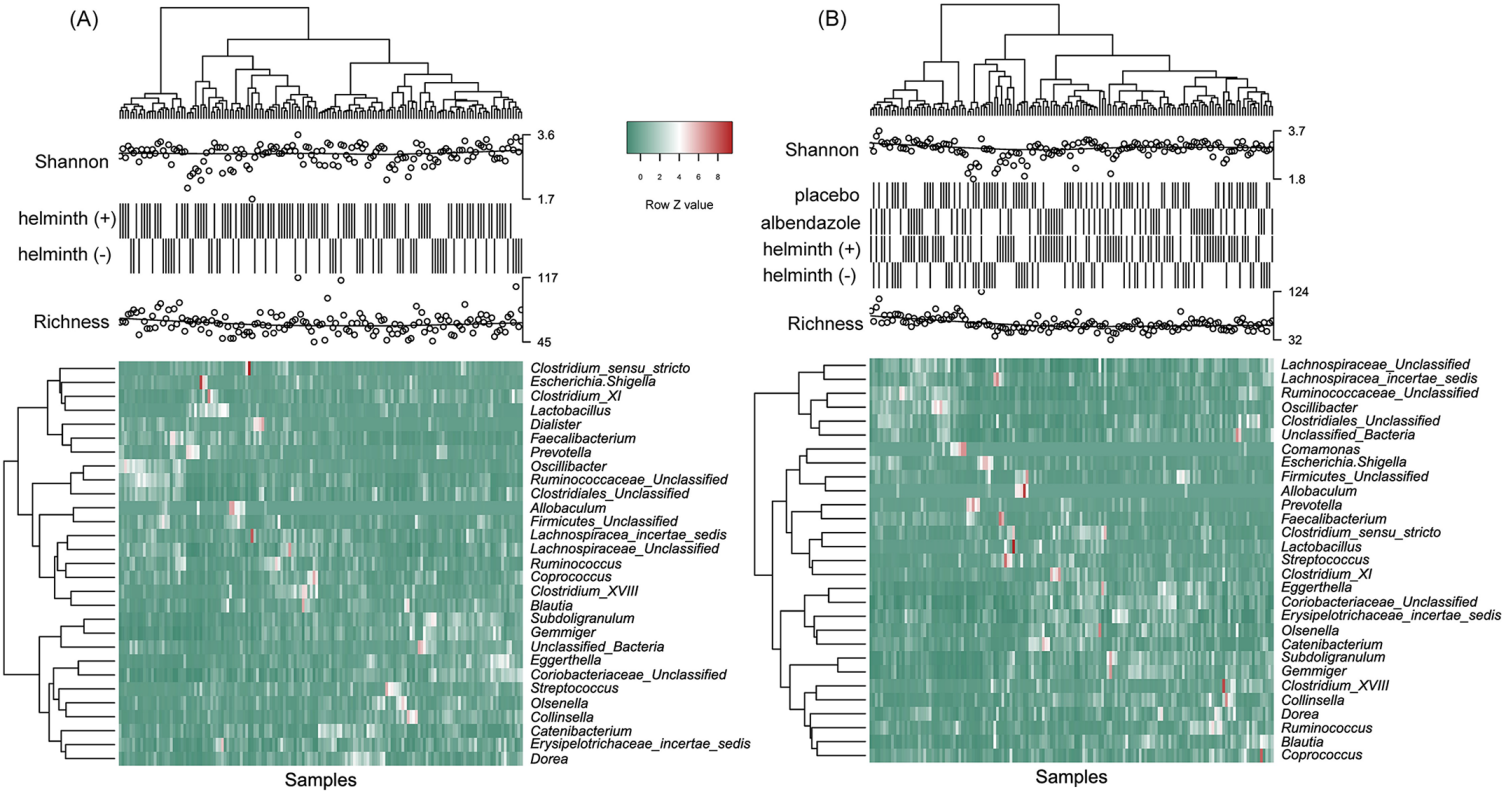
Heatmaps showing the relative abundance of the 29 most abundant genera of each sample at pre-treatment (A) and post-treatment (B). Each column in the heatmap represents a specific sample and each row represents a genera. Colors represent the scaled relative abundance of genera with green and red representing low and high abundance, respectively. Samples and genera were clustered hierarchically (using the Ward method [38]) based on Euclidean distance of the relative abundance profiles and were depicted on the top and left dendrogram, respectively. The infection status, treatment allocation, Shannon and richness indices for all samples were annotated above the heatmap. Circles in Shannon and richness represents the diversity indices for each sample. There is no clustering of samples or genera based on infection or treatment status.

The average relative abundances of all bacterial genera at both time-points were below 10%, with the highest being in the phylum Firmicutes, specifically the genus *Catenibacterium* (6.7% at pre-treatment) and the unclassified genus belonging to the family Ruminococcaceae (5.6% at post-treatment). The relative abundance at the genus level as well as the dominant genera vary between populations as observed in studies where samples in rural Ecuador [20] or Malaysia were compared with the US [21] or in studies where samples of healthy European and American adults were analysed [37]. To illustrate the bacterial genera profile in relation to infection and treatment status, we selected the 29 genera (at pre and post-treatment) with an average of relative abundance across all samples larger than 1%. Genera from phylum Firmicutes are the most dominant (21 of 29 genera belongs to Firmicutes). As shown in heatmaps based on composition of the most prevalent genera, no significant clustering could be seen, neither at the level of bacteria nor at the level of individuals (Fig 3A and 3B) in relation to helminth infection or treatment, which indicates that neither helminths nor treatment affected the predominant genera in the gut.

### The association of infection and treatment with the microbiome composition at the phylum level

**Fig 4:**
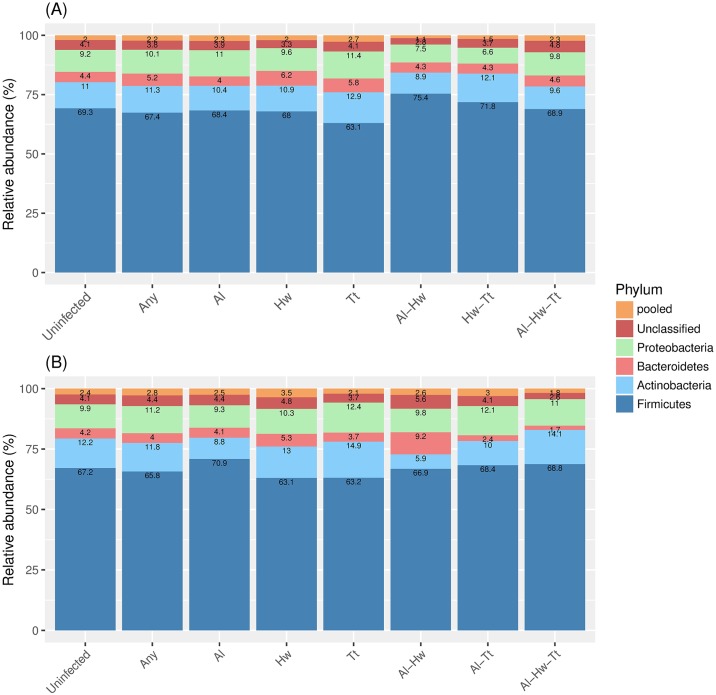
The microbiome composition at pre-treatment (A) and post-treatment (B) stratified by helminth infection. The stacked bar plots represent the relative abundance for each of the most abundant phyla where the Unclassified represents the category of sequences that could not be assigned to a phyla, and the pooled category consists of the remaining 13 phyla with average relative abundance less than 1%. The numbers inside the stacked bar plots show the relative abundance of the specific taxa. The microbiome compositions were depicted for group of helminth-uninfected (Uninfected), any helminth infected (Any), single helminth infection (*A*. *lumbricoides* (Al), hookworm (Hw) or *T*. *trichiura* (Tt)), double infection (Al—Hw, Al—Tt and Hw—Tt) or triple infections (Al—Hw—Tt).

Using the Dirichlet-multinomial regression model, we observed that there was no difference on the microbiome composition at the phylum level when subjects with any helminth infection were compared with uninfected ones either at pre (Fig 4A) or at post treatment (Fig 4B) time points. The same was the case when infection with a specific helminth species was considered (Fig 4A and 4B).

The Dirichlet-multiomial regression model was also used to discern the effect of helminths and treatment on the microbiome data at post treatment. Six bacterial categories were considered in the analyses with Firmicutes used as a reference. The effect of treatment on microbiome composition in all individuals irrespective of whether they were infected or not at post-treatment was not significant. No differences were observed between placebo and albendazole at post-treatment (*p*-value = 0.305, Table 2A, likelihood ratio test).

**Table 2:** The association between each bacteria phylum with treatment and infection at post-treatment.

Predictor			N	OR (95% CI)					*p*-values
				Actinobacteria	Bacteroidetes	Proteobacteria	Unclassified	Pooled	
(A)									
placebo			81	Reference					
albendazole			69	1.15 (0.91,1.45)	0.97 (0.70,1.35)	1.18 (0.92,1.51)	0.94 (0.68,1.29)	1.11(0.78,1.58)	0.305
(B)									
placebo	infected	(group 4)	34	Reference					
	uninfected	(group 2)	13	0.95 (0.62,1.46)	**0.49 (0.27,0.91)**	0.71 (0.45,1.10)	0.80 (0.45,1.43)	**0.47 (0.23,0.96)**	0.069
albendazole	infected	(group 3)	13	**1.57 (1.05,2.35)**	**0.35 (0.18,0.70)**	1.01 (0.66,1.55)	0.76 (0.41,1.38)	0.75 (0.39,1.47)	**0.004**
	uninfected	(group 1)	34	1.18 (0.54,2.57)	0.79 (0.24,2.54)	0.89 (0.40,1.10)	0.79 (0.27,2.36)	0.83 (0.25,2.74)	0.371

The estimated OR (odds ratio) and 95% CI (confidence interval) were obtained from the Dirichlet—multinomial regression and the *p*-values were obtained from the likelihood ratio test. Firmicutes is used as a reference for bacterial category. (A) The regression was fitted on all subjects irrespective of their infection status to assess the significant effect of treatment on microbiome composition. (B) The regression was fitted on subjects who were infected at baseline (N = 94) to assess the significance of microbiome composition of each group compared to placebo infected (group 4). Bold represents the significant association between specific bacteria phylum with predictors. Bold represents the significant association. OR: odds ratio, CI: confidence interval.

**Fig 5:**
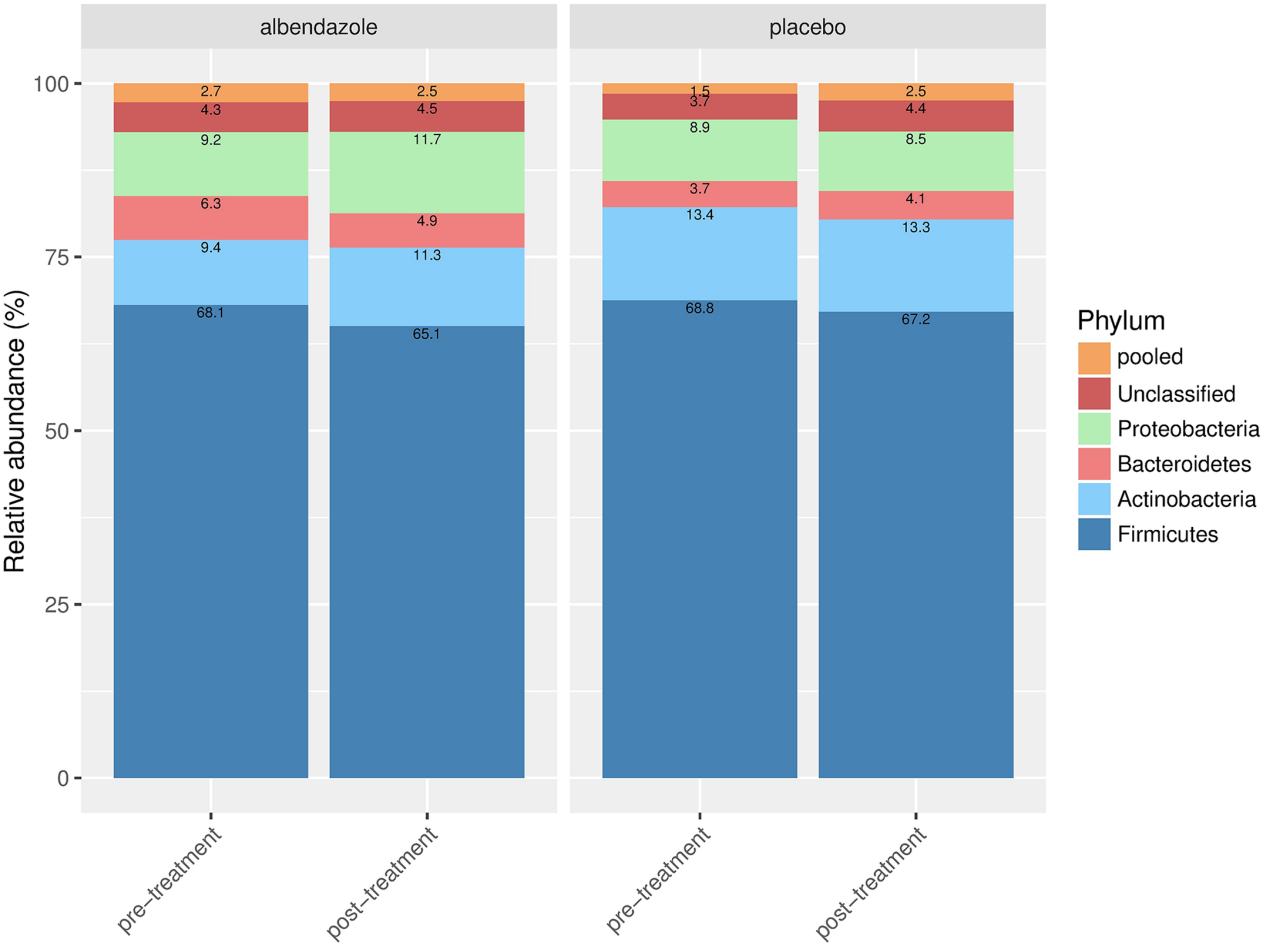
Direct treatment effect on microbiome composition. Details for the stacked barplots were as given in Fig 4. The microbiome composition is shown at pre and post-treatment for subjects who were uninfected at pre-treatment and remained uninfected at post-treatment in albendazole and placebo arm.

We further selected subjects who were infected at baseline (N = 94) and characterized their microbiome composition at post-treatment with regard to their infection status and treatment arm, namely: subjects who lost their infection either in the albendazole (group 1, N = 34) or placebo arm (group 2, N = 13), and subjects who remained infected in either the albendazole (group 3,N = 13) or placebo arm (group 4, N = 34). We compared the microbiome composition of the first three groups to the group of remained infected in the placebo arm (group 4) as the latter group were neither influenced by treatment nor the changing of infection status. When subjects who were infected at pre-treatment and lost their infection in the albendazole arm were compared to subjects who remained-infected in placebo group, no differences were observed (*p*-value of 0.371, Table 2B), indicating that removing helminths with albendazole did not change the microbiome profile at a phylum level. Furthermore, in subjects who lost their infection in the placebo arm, there was a trend for decrease in Bacteroidetes and pooled category (OR 0.49, 95% CI:(0.27,0.91) and OR 0.47, 95% CI:(0.23,0.96), respectively, Table 2B), moreover, the whole composition in this group did not differ significantly from that in the group of remained infected in the placebo arm (*p*-value of 0.069). These two comparisons suggest that removing helminths regardless of treatment did not alter the microbiome composition when analysed at a phylum level. Interestingly, the comparison of microbiome composition between subjects who remained infected in the albendazole group was significantly different from the microbial composition in subjects who remained infected in the placebo group (*p*-value of 0.004,Table 2B). This difference was driven by the increasing odds of having Actinobacteria (OR 1.57, 95% CI of (1.05, 2.35)) and the decreasing odds of having Bacteroidetes (OR 0.35, 95% CI: (0.18,0.70)). To further analyse the direct treatment effect without the influence of helminth infection, we selected subjects who were uninfected at baseline and remained-uninfected at post-treatment (N = 44). For these subjects, we compared the microbial composition at post-treatment of subjects who received albendazole versus those who received placebo. No difference was observed (the estimate odds ratios range from 0.88, 95% CI: (0.56, 1.39) to 1.42, 95% CI: (0.88, 2.29), *p*-value = 0.666, illustrated in Fig 5), indicating that albendazole alone does not seem to affect the microbiome composition in uninfected subjects when compared at a phylum level.

As neither treatment alone nor the infection affected the microbial composition, we further hypothesized that the significant difference in microbiome composition in subjects who remained infected and received albendazole compared to the group that remained infected in the placebo arm was caused by the alteration of the abundance of Actinobacteria and Bacteroidetes during the treatment period. To test this hypothesis, we used the inverted beta-binomial test to compare the relative abundance of Actinobacteria and Bacteroidetes in subjects who remained infected in albendazole group at pre-treatment to the relative abundances of these bacterial phyla at post-treatment. While the relative abundance of Actinobacteria did not change significantly between pre and post-treatment (*p*-value of 0.155, inverted beta binomial test), the relative abundance of Bacteroidetes was estimated to be 1.88 fold higher at pre-treatment compared to post-treatment (*p*-value of 0.012, inverted beta binomial test). This result indicates that there is a complex interaction between helminths and treatment, which induces a change in bacterial composition during the treatment period. Using the same analysis, the direct effect of albendazole was assessed by comparing subjects who were uninfected but received albendazole at pre treatment and remained uninfected at post treatment. Although some differences were seen in the microbiome composition between pre and post-treatment, specifically in the phyla Actinobacteria, Bacteroidetes and Proteobacteria, these differences were not statistically significant (*p*-values of 0.149, 0.267 and 0.064, respectively). This is in line with the finding when we used the Dirichlet-multinomial regression model where no direct effect of albendazole on the microbiome composition was found. In addition, similar microbiome composition was seen in subjects free of helminth infection at baseline who received placebo and remained uninfected at post-treatment, which suggests that the microbiome was stable over time.

### The association of Bacteroidetes genera with infection and treatment

In the Dirichlet—multinomial regression analysis carried out at the phylum level, Bacteroidetes was the phyla that showed significant differences in subjects who remained infected in the albendazole arm compared to those who remained infected in the placebo arm. We dissected this further to assess which Bacteroidetes genera accounted for this difference using the Dirichlet-multinomial regression model on 6 bacterial categories which were obtained as follows. The phylum Bacteroidetes was divided into three categories, namely the *Bacteroides*, *Prevotella* and pooled Bacteroidetes. The first two genera were chosen as they were the two most abundant in the phylum Bacteroidetes. In the analyses, as 6 categories are needed, we included another three phyla, i.e., Actinobacteria, Firmicutes and pooled remaining phyla (pooled Phyla). As for the modelling at the phylum level, Firmicutes was used as a reference. Similar to the analyses at the phylum level, we characterized the association of infection and treatment on these 6 bacterial categories that comprised the genera belonging to Bacteroidetes.

When considering the whole study subjects irrespective of infection status, there was no difference between albendazole and placebo (Table 3A). When 94 infected subjects at pre-treatment were selected and 6 bacterial categories as above were analysed with regard to infection and treatment, we observed a decrease in odds of having *Prevotella* in subjects who lost their helminth infection in placebo group (OR 0.44, 95% CI: (0.21,0.90)) compared to subjects who remained infected in placebo group although this fell short of statistically significant (*p*-value of 0.086, Table 3B). Furthermore, in line with the finding at the phylum level, we also observed a significant difference in microbial composition of subjects who remained infected with albendazole compared to the microbial composition of subjects who remained infected in the placebo group (*p*-value of 0.016). This alteration was mainly due to the increase in odds of having Actinobacteria (OR 1.54, 95% CI: (1.00, 2.35)) and a decrease in odds of having *Prevotella* (OR 0.44, 95% CI: (0.21, 0.94)), suggesting that the decrease in Bacteroidetes at the phylum level observed in Table 2B was driven by *Prevotella*.

**Table 3:** The association between combination of bacteria taxa with treatment and infection at post-treatment.

Predictor			N	OR (95% CI)					*p*-values
				Actinobacteria	Bacteroidetes			pooled Phyla	
					*Bacteroides*	*Prevotella*	pooled Bacteroidetes		
(A)									
placebo			81	Reference					
albendazole			69	1.16 (0.90,1.49)	1.00 (0.61,1.62)	1.02 (0.69,1.50)	0.97 (0.65,1.45)	1.08 (0.85,1.37)	0.7
(B)									
placebo	infected	(group 4)	34	Reference					
	uninfected	(group 2)	13	0.92 (0.59,1.43)	0.49 (0.18,1.34)	**0.44 (0.21,0.90)**	0.49 (0.24,1.03)	0.73 (0.48,1.10)	0.086
albendazole	infected	(group 3)	13	**1.54 (1.00,2.35)**	0.79 (0.32,1.97)	**0.44 (0.21,0.94)**	0.40 (0.18,0.89)	0.92 (0.61,1.40)	**0.016**
	uninfected	(group 1)	34	1.17 (0.51,2.67)	0.78 (0.15,4.13)	0.78 (0.21,2.93)	0.74 (0.18,3.00)	0.83 (0.38,1.80)	0.478

The Dirichlet—multinomial regression was fitted on 6 categories consists of three genera under Bacteroidetes phyla and three phyla (Actinobacteria, Firmicutes and pooled Phyla). Here, the pooled Phyla consists of *Proteobacteria*, Unclassified and Pooled category as in Table 2. Firmicutes is used as a reference. Similar as in Table 2, (A) the regression was fitted on all subjects irrespective of their infection status to assess the significance effect of treatment on the bacterial composition. (B) The regression was fitted on infected subjects at baseline (N = 94 to assess the significance of each group compared to placebo infected (group 4). Bold represents the significant association. OR: odds ratio, CI: confidence interval.

## Discussion

There are two unique aspects to the current study on the effect of helminths on the gut microbiome in subjects living in rural areas of Indonesia, namely the combination of the study design and the statistical approach. The statistical parametric or nonparametric approaches are typically used to test the hypothesis whether the microbiome compositions are significantly different between groups [20–23]. While the nonparametric approach suffers from lack of statistical power when the sample size is small [39], available parametric approaches consider the abundance of each bacterial categories separately, hence requiring multiple testing corrections. The previous studies in Zimbabwe, Malaysia and Ecuador relating microbiome and helminths compared the difference of abundance of certain bacteria category between groups by using the standard or paired *t*-test and addressed multiple testing by Bonferonni corrections or False Discovery Rate [20–22]. The clustering of bacteria has been investigated before using descriptive nonparametric approaches such as PCA or NMDS. When we applied these method to our genera data, no clustering was observed; neither by infection status nor by randomization arm. This might be an indication that PCA or NMDS were unable to capture the correlation between genera. We further analysed the multivariate data composed of 6 phyla (Firmicutes, Actinobacteria, Bacteroidetes, Proteobacteria, Unclassified bacteria and pooled category) simultaneously in relation to helminth infection status and treatment using a parametric approach. This multivariate approach takes into account the nature of metagenomics data, such as the abundance of all phyla forming the compositional structure and that these abundances are known to vary highly between subjects [40]. Our method is able to quantify the relationship between the whole bacteria community with regard to the presence/absence of helminths or antihelminthic treatment while taking into account the correlational structure between bacterial categories imposed by the compositional nature. As bacterial categories are correlated, the decrease of one category should cause the increase of other categories and *vice versa* [7, 41]. Several microbiome studies have reported the change of the ratio Firmicutes to Bacteroidetes [42, 43]. Thus, inference with regard to the decrease or increase of certain bacteria only makes sense when all bacterial categories are considered.

The reparameterization of Dirichlet—multinomial in the data analyses provided an interpretation in terms of odds ratios on how bacterial categories were affected by the helminth infection or treatment allocation. To obtain odds ratios, a reference category needs to be selected. In this study, we used Firmicutes as a reference due to its high abundance among bacterial categories as well as its presence in all samples. The high abundance of Firmicutes remained relatively stable, which had the advantage of allowing us to reveal subtle differences in other bacterial categories.

One potential limitation of our multivariate method is that the number of bacterial categories to be modelled was limited. As a consequence, taxa had to be pooled. Such a procedure assumes that the effect of the underlying taxa are captured in one single parameter. On the other hand, pooling can be viewed as a practical way to deal with sequencing error by providing a more robust model [26]. Instead of pooling, one might use a shrinkage method as proposed by Chen and Li [26] to deal with multiple rare taxa. As an alternative to biostatistical regression methods, machine learning methods are typically used for analysis of microbiome data. However, such methods require larger samples to allow the split into a training and a validation set. Our dataset is too small for such a method. Moreover, this method ignores the correlation structure, such as overdispersion.

It should be noted that the coverage depth in our study is relatively low (in average of 6000 reads per sample) as a result of using pyrosequencing platform (454) compared to more recent deep sequencing technologies (Illumina). We noted that two microbiome studies have reported similar average reads per samples as in our study [20, 44]. As a consequence, rare taxa or taxa with low abundance might not be detected [45], and it is also possible that the similar diversity that we observed could be caused by the use of this platform. However, a direct comparison between Illumina MiSeq and the 454 platform has revealed that the limitation of the 454 is at the genus and family level, while at the higher taxonomic level (such as order, class and phylum level), the 454 platform is able to detect the same number of bacterial categories as the Illumina platform [30]. This could be considered as an advantage of this approach allowing the analysis at the phylum level.

Another unique aspect regarding our study was that a placebo-controlled anthelminthic trial design was used, while other studies were either observational or used an intervention without a placebo group. A control group that did not get the anthelminthic treatment (received placebo) has the advantage of controlling for confounders and estimating a direct treatment effect [46, 47].

There were no significant differences in the microbiome composition, analyzed at the phylum level, of subjects with and without helminth infection at baseline, nor at the 21 months time point. One possibility is the low resolution of the bacterial data at phylum level. It is also possible that the similarity in microbiome composition between infected and uninfected subjects is due to infection history[21]. Surprisingly, we observed a significant difference in the microbiome composition between placebo and albendazole-treated subjects at post-treatment in those who remained infected (Table 2B). This difference seemed to be represented by an increase in relative abundance of Actinobacteria and a decrease in relative abundance of Bacteroidetes. This difference in the relative abundance of Bacteroidetes was confirmed by comparing paired samples at pre and post-treatment in the albendazole group who were infected at baseline and remained infected at post-treatment. No significant difference in microbiome composition was found when comparing the albendazole and placebo arms in subjects who remained uninfected, or when comparing pre and post-treatment in those who received albendazole but remained uninfected. These data indicate that first of all, microbiome composition is stable over time and second, albendazole has no direct effect on microbiome composition. Together, our results suggest that the interplay between anthelminthic treatment and helminths in the gut has a complex effect on the microbiome composition. We observed that deworming is more effective against certain helminth species but not others. Indeed, *T*. *trichiura* infection was dominant after treatment in our study. This means that infected subjects who had received placebo harboured different helminth species than those who had received albendazole. However, at pre-treatment, there was no difference between the microbiome associated specifically with *T*. *trichiura*, *A*. *lumbricoides* or hookworm and therefore the effect of albendazole on the microbiome at post-treatment in infected subjects can not only be due to the dominance of *T*. *trichiura* but possibly the result of a combination of *Trichuris* and albendazole on the microbiome composition. It should be noted that in a recent study taking a different approach from us by using machine learning techniques, considering all taxonomic levels, and large sample size from not only Indonesia but also Liberia, differences in certain taxa were found to be worm-specific [30]. Therefore, to confirm whether *T*. *trichiura* has a different effect on microbiome composition after albendazole treatment compared to other helminth species, further and larger studies are needed.

With regard to the treatment effect, a study in Malaysia reported the increasing abundance of Bacteroidales (an order of Bacteroidetes) and the decreasing abundance of Clostridiales (an order of Firmicutes) after treatment [23]. This result might be confounded as there was no control group to assess the treatment effect. Another interventional study was carried out in Zimbabwe, but it did not provide information on the effect of treatment in those who remained infected since the microbiome composition was only measured in subjects who completely cleared their helminths.

A longitudinal setting in microbiome studies has the advantage of analysing the microbiome composition at different time points in the same population. However, the studies using longitudinal approach differed in the length of follow-up time. The studies in Malaysia [23] had a follow-up time of 21 days, the study in Zimbabwe [22] examined the microbiome composition at 12 weeks after treatment while our study had the longest follow-up time of 21 months (with treatment given every three months). Thus, so far the previous studies have examined the effect of short term removal of helminths on microbiota [23], while in our study, we used a longer follow-up time to ensure succesful and long lasting deworming of the subjects. Differences in study design and techniques used for collection and analysis of samples hamper comparison across studies.

The regression model used in this study is only applicable in a cross-sectional manner and assumes a simple correlation structure between bacterial categories. Such a method could be extended to more complex correlation structures. One is the correlation between bacterial categories or between the microbiome composition of the same subject measured at different time points. A statistical test for paired two categorical counts is available, however to model the change in microbiome composition over time we would need to extend our model.

To conclude, the microbiome composition is likely to change due to interactions between helminth and anthelminthic treatment, but a direct impact of treatment on microbiome composition has not been observed. Larger studies are needed to dissect these effects of treatment and also to take into account the history of helminth infection. Furthermore, new statistical methods that allow longitudinal analysis of changes in the microbiome composition need to be developed.

### Availability of data and materials

The 16S rDNA assembled sequences, annotation and abundances from all the Indonesia samples are available for download from Nematode.net (nematode.net/Indonesia_Microbiome.html) [48].”

## Supporting information

S1 Fig Bacterial diversity in relation with helminth at pre and post-treatment. The Shannon (top) and richness (bottom) indices were computed in helminth infected (Helminth (+)) and uninfected (Helminth (-)) subjects.
(TIF)

S1 Table List of primers and probe sequences used in detecting the helminth species. (DOCX)

S2 Table The characteristics of participants of current study and total population in Nangapanda. (DOCX)
