# Supplementary figures and images for: Dynamic changes in human-gut microbiome in relation to a placebo-controlled anthelminthic trial in Indonesia
Source: PLoS Negl Trop Dis. 2018 Aug 9;12(8):e0006620. doi: 10.1371/journal.pntd.0006620 (PMC6084808; doi:10.1371/journal.pntd.0006620)

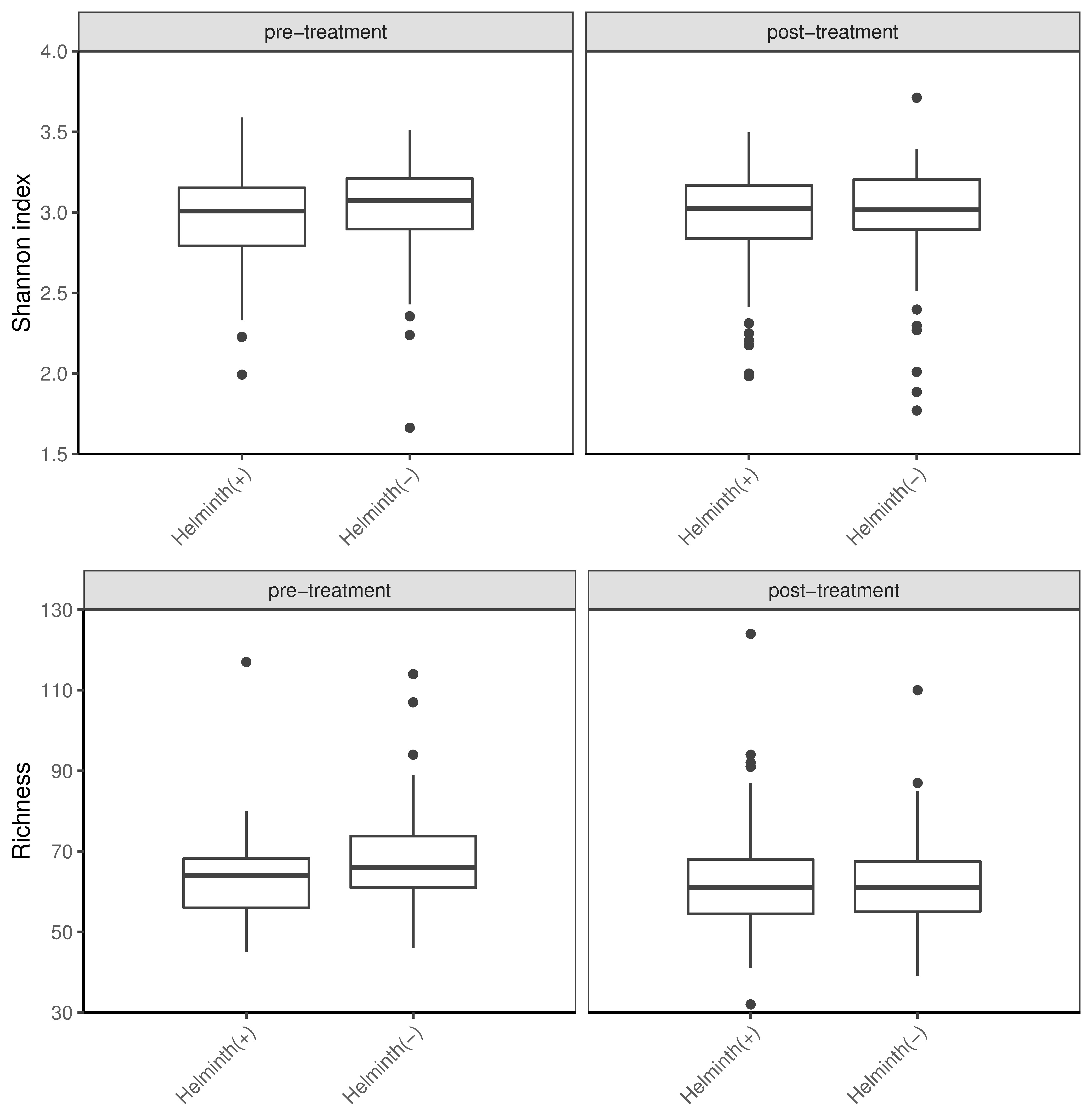

Supplement: S1 Fig — The Shannon (top) and richness (bottom) indices were computed in helminth infected (Helminth (+)) and uninfected (Helminth (-)) subjects. (TIF) [file pntd.0006620.s001.tif]
