# Supplementary material for: Dynamic changes in human-gut microbiome in relation to a placebo-controlled anthelminthic trial in Indonesia
Source: PLoS Negl Trop Dis. 2018 Aug 9;12(8):e0006620. doi: 10.1371/journal.pntd.0006620 (PMC6084808; doi:10.1371/journal.pntd.0006620)
Supplement: S1 Table — (DOCX) [file pntd.0006620.s002.docx]

**S1 Table. List of primers and probe sequences used in detecting the helminth species**

| **STH species** | **Oligonucleotide name** | **Oligonucleotide sequences** | **References** |
| --- | --- | --- | --- |
| *Ascaris lumbricoides* | Alum_96F | 5’-GTAATAGCAGTCGGCGGTTTCTT-3’ | Wiria, *et al.* 2010 [24] |
|  | Alum_183R | 5’-GCCCAACATGCCACCTATTC-3’ |  |
|  | Alum_124T_YY | Yakima Yellow-5’-TTGGCGGACAATTGCATGCGAT-3’ |  |
| *Necator americanus* | Na_58F | 5’-CTGTTTGTCGAACGGTACTTGC-3’ | Wiria, *et al.* 2010 [24] |
|  | Na_158R | 5’-ATAACAGCGTGCACATGTTGC-3’ | Verweij, *et al.* 2007 [28] |
|  | Na_81T_FAM | FAM-5’-CTGTACTACGCATTGTATAC-3’ |  |
| *Ancylostoma duodenale* | Ad_125F | 5’-GAATGACAGCAAACTCGTTGTTG-3’ | Wiria, *et al.* 2010 [24] |
|  | Ad-195R | 5’-ATACTAGCCACTGCCGAAACGT-3’ | Verweij, *et al*. 2007 [28] |
|  | Ad_155_TR | Texas Red-5’-ATCGTTTACCGACTTTAG-3’ |  |
