# Supplementary material for: Dynamic changes in human-gut microbiome in relation to a placebo-controlled anthelminthic trial in Indonesia
Source: PLoS Negl Trop Dis. 2018 Aug 9;12(8):e0006620. doi: 10.1371/journal.pntd.0006620 (PMC6084808; doi:10.1371/journal.pntd.0006620)
Supplement: S2 Table — (DOCX) [file pntd.0006620.s003.docx]

**S2Table. The characteristics of participants of current study and total population in Nangapanda.**

|  | **Participants of current study** | | | | **Whole Study Population** | | | |
| --- | --- | --- | --- | --- | --- | --- | --- | --- |
| Characteristics | albendazole | | placebo | | albendazole | | placebo | |
|  | N | Result | N | Result | N | Result | N | Result |
| Sex, female | 69 | 39 (56.5) | 69 | 45 (55.6) | 1729 | 920 (53.2) | 1762 | 971 (55.1) |
| Age, mean(sd) | 69 | 27.38 (16.52) | 81 | 27.85 (16.91) | 1729 | 27.96 (18.99) | 1762 | 27.89 (18.82) |
| Z-score of BMI (≤ 18 years old) (mean, SD) | 28 | -1.35 (1.17) | 33 | -0.86 (0.96) | 386 | - 1.39 (1.25) | 427 | - 1.23 (1.16) |
| BMI (> 19 years old) (mean, SD) | 41 | 21.99 (3.3) | 48 | 23.8 (4.28) | 582 | 21.79 (3.61) | 575 | 22.28 (3.98) |
| **Clinical Symptoms (n,%)** |  |  |  |  |  |  |  |  |
| Diarrhea | 69 | 0 (0) | 81 | 0 (0) | 995 | 4(0.4) | 1019 | 2 (0.2) |
| Fever | 69 | 0 (0) | 81 | 1 (1.2) | 995 | 15 (1.5) | 1019 | 22 (2.2) |
| Malaise | 69 | 1 (1.4) | 81 | 2 (2.5) | 995 | 25 (2.5) | 1019 | 29 (2.8) |
| **Social Economics (n,%)** |  |  |  |  |  |  |  |  |
| **House Material (>75%)** | 69 |  | 81 |  | 1719 |  | 1744 |  |
| Stone |  | 20 (29.0) |  | 24(29.6) |  | 461 (26.8) |  | 452 (25.9) |
| Wood |  | 9 (13.0) |  | 9 (11.1) |  | 292 (17.0) |  | 322(18.5) |
| Bamboo/Triplex |  | 40(58.0) |  | 48 (59.3) |  | 966 (56.2) |  | 970 (55.6) |
| **Processed Water** | 69 | 39 (56.5) | 81 | 40 (49.4) | 1719 | 967 (56.3) | 1744 | 919 (52.7) |
| **Toilet** | 69 |  | 81 |  | 1719 |  | 1744 |  |
| Indoor |  | 52 (75.4) |  | 59 (72.8) |  | 1029 (59.9) |  | 1018 (58.4) |
| Outdoor |  | 17 (24.6) |  | 22 (27.2) |  | 690 (40.1) |  | 726 (41.6) |
| **Floor material** | 69 |  | 81 |  | 1719 |  | 1744 |  |
| Ceramic |  | 10 (14.5) |  | 9 (11.1) |  | 201 (11.7) |  | 195 (11.2) |
| Cement |  | 39 (56.5) |  | 53 (65.4) |  | 1211 (70.4) |  | 1257 (72.1) |
| Sand |  | 18 (26.1) |  | 18 (22.2) |  | 277 (16.1) |  | 257 (14.7) |
| Other |  | 2 (2.9) |  | 1 (1.2) |  | 30 (1.7) |  | 35 (2.0) |
| **Kitchen** | 69 |  | 81 |  | 1719 |  | 1744 |  |
| Inside |  | 40 (58.0) |  | 47 (58.0) |  | 977 (56.8) |  | 949 (54.4) |
| Outside |  | 29 (42.0) |  | 34 (42.0) |  | 742 (43.2) |  | 795 (45.6) |
| **Managing Household waste** | 69 |  | 81 |  | 1719 |  | 1744 |  |
| Throw to waste pipeline |  | 4 (5.8) |  | 12 14.8) |  | 202 (11.8) |  | 229 (13.1) |
| Throw to the river |  | 8 (11.6) |  | 5 (6.2) |  | 147 (8.6) |  | 183 (10.5) |
| Throw it near the house |  | 55 (79.7) |  | 60 (74.1) |  | 1343 (78.1) |  | 1306 (74.9) |
| Other |  | 2 (2.9) |  | 4 (4.9) |  | 27 (1.6) |  | 26 (1.5) |
| **Washing hands before eating** | 49 |  | 57 |  | 1147 |  | 1114 |  |
| Yes |  | 34 (69.4) |  | 40 (70.2) |  | 717 (62.5) |  | 674 (60.5) |
| No |  | 15 (30.6) |  | 17 (29.8) |  | 430 (37.5) |  | 440 (39.5) |
| **Washing hands after defecate** | 49 |  | 56 |  | 1143 |  | 1119 |  |
| Yes |  | 35 (71.4) |  | 41 (73.2) |  | 790 (69.1) |  | 757 (67.6) |
| No |  | 14 (28.6) |  | 15 (26.8) |  | 353(30.9) |  | 362 (32.4) |
| **Toilet use for defecation** | 48 |  | 55 |  | 1131 |  | 1096 |  |
| Yes |  | 41 (83.7) |  | 50 (90.9) |  | 921(81.4) |  | 888 (81.0) |
| No |  | 7 (14.3) |  | 5 (9.1) |  | 210 (18.6) |  | 208 (19.0) |
| **Diet (n,%)** |  |  |  |  |  |  |  |  |
| **Staple Food (two mostly eaten)** | 21 |  | 23 |  | 468 |  | 502 |  |
| Rice |  | 18 (85.7) |  | 22 (95.7) |  | 362 (72.4) |  | 423 (84.3) |
| Cassava |  | 17 (81.0) |  | 19(82.6) |  | 304 (65.0) |  | 362 (72.1) |
| **Main Food (two mostly eaten)** | 21 |  | 23 |  | 468 |  | 502 |  |
| Fish |  | 19 (90.5) |  | 21(91.3) |  | 448 (95.7) |  | 480 (95.6) |
| Egg |  | 14 (66.7) |  | 19 (82.6) |  | 368 (78.6) |  | 418 (83.3) |

The number of subject (n) of the total participants who were surveyed (N).
